# An Investigation of Bilateral Symmetry During Manual Wheelchair Propulsion

**DOI:** 10.3389/fbioe.2015.00086

**Published:** 2015-06-11

**Authors:** Shelby L. Soltau, Jonathan S. Slowik, Philip S. Requejo, Sara J. Mulroy, Richard R. Neptune

**Affiliations:** ^1^Department of Mechanical Engineering, The University of Texas at Austin, Austin, TX, USA; ^2^Pathokinesiology Laboratory, Rancho Los Amigos National Rehabilitation Center, Downey, CA, USA; ^3^Rehabilitation Engineering, Rancho Los Amigos National Rehabilitation Center, Downey, CA, USA

**Keywords:** asymmetry, side-to-side differences, hand dominance, speed, graded, biomechanics

## Abstract

Studies of manual wheelchair propulsion often assume bilateral symmetry to simplify data collection, processing, and analysis. However, the validity of this assumption is unclear. Most investigations of wheelchair propulsion symmetry have been limited by a relatively small sample size and a focus on a single propulsion condition (e.g., level propulsion at self-selected speed). The purpose of this study was to evaluate bilateral symmetry during manual wheelchair propulsion in a large group of subjects across different propulsion conditions. Three-dimensional kinematics and handrim kinetics along with spatiotemporal variables were collected and processed from 80 subjects with paraplegia while propelling their wheelchairs on a stationary ergometer during three different conditions: level propulsion at their self-selected speed (free), level propulsion at their fastest comfortable speed (fast), and propulsion on an 8% grade at their level, self-selected speed (graded). All kinematic variables had significant side-to-side differences, primarily in the graded condition. Push angle was the only spatiotemporal variable with a significant side-to-side difference, and only during the graded condition. No kinetic variables had significant side-to-side differences. The magnitudes of the kinematic differences were low, with only one difference exceeding 5°. With differences of such small magnitude, the bilateral symmetry assumption appears to be reasonable during manual wheelchair propulsion in subjects without significant upper-extremity pain or impairment. However, larger asymmetries may exist in individuals with secondary injuries and pain in their upper extremity and different etiologies of their neurological impairment.

## Introduction

Manual wheelchair propulsion is commonly assumed to be a symmetric task. The rationale for this assumption is that any asymmetry, combined with the uncoupled nature of the wheels, would make straight-line propulsion difficult (e.g., de Groot et al., 2002). Resulting steering corrections could lead to increased energy cost and other unfavorable effects (e.g., Vegter et al., 2013a), and therefore experienced manual wheelchair users likely develop symmetrical propulsion mechanics over time.

However, the prevalence of the symmetry assumption has also been influenced by the limitations in available data collection systems. Early single-camera systems only allowed the measurement of unilateral kinematics that were usually restricted to the sagittal plane (e.g., Sanderson and Sommer, 1985; Masse et al., 1992; Veeger et al., 1992). Experimental set-ups involving mirrors and/or an additional camera allowed measurement of frontal plane kinematics and the calculation of 3D kinematics (e.g., van der Woude et al., 1989; Veeger et al., 1989; Goosey et al., 1998). The collection of bilateral 3D kinematics (e.g., Rao et al., 1996; Shimada et al., 1998) eventually became standard with the proliferation of multi-camera systems. By this time, instrumented wheels and other devices that allow the measurement of handrim kinetics had also been developed (e.g., Asato et al., 1993; Rodgers et al., 1994; Wu et al., 1998). However, many current laboratories are equipped with only one instrumented wheel due to the high cost of these devices (e.g., Hurd et al., 2008a). Thus, bilateral measurements often require multiple trials in which the instrumented wheel is switched back and forth between sides, effectively doubling the time and effort necessary for data collection.

Even with bilateral data collection, studies often do not report results for both sides, but elect to either average the data across both limbs (e.g., Boninger et al., 2000) or select only one limb for analysis (e.g., Finley et al., 2004; Mercer et al., 2006; Gagnon et al., 2014). Among the studies that have examined side-to-side differences in propulsion mechanics, there is a lack of consensus regarding the presence of asymmetry. Some studies have suggested that there is no significant asymmetry in kinematic (e.g., Goosey and Campbell, 1998), kinetic (e.g., Hurd et al., 2008b), or spatiotemporal (e.g., de Groot et al., 2002) variables. However, others have found significant side-to-side differences in similar propulsion variables (Hurd et al., 2008a; Stephens and Engsberg, 2010). The lack of statistically significant differences in most previous studies may be due to small sample sizes (*n* ≤ 20). In addition, studies have suggested that asymmetry may be present in specific individuals even if it is not detectable when comparing side-to-side group averages (e.g., Koontz et al., 2001; Schnorenberg et al., 2014).

Another limitation of previous studies is most have only examined side-to-side differences during one propulsion condition (e.g., level propulsion at self-selected speed). However, recent studies have suggested that the level of asymmetry may be influenced by the terrain (Hurd et al., 2008a, 2009). The purpose of this study was to evaluate bilateral symmetry during manual wheelchair propulsion in a large number of subjects across different propulsion conditions. These results have important implications for experimental setups in future analyses of wheelchair propulsion mechanics.

## Materials and Methods

### Subjects

Symmetry data were collected and analyzed from 80 individuals with paraplegia who were free of shoulder pain and used a manual wheelchair at least 50% of the time for community mobility (74 men, 6 women; age: 37.0 ± 9.9 years; time from injury: 9.0 ± 6.6 years; height: 1.72 ± 0.09 m; mass: 74.5 ± 16.9 kg). Dominant side was self-reported by each subject (74 right-handed, 6 left-handed). The participants were recruited from outpatient clinics at Rancho Los Amigos National Rehabilitation Center and provided written informed consent in accordance with the Institutional Review Board.

### Data collection

**Figure 1:**
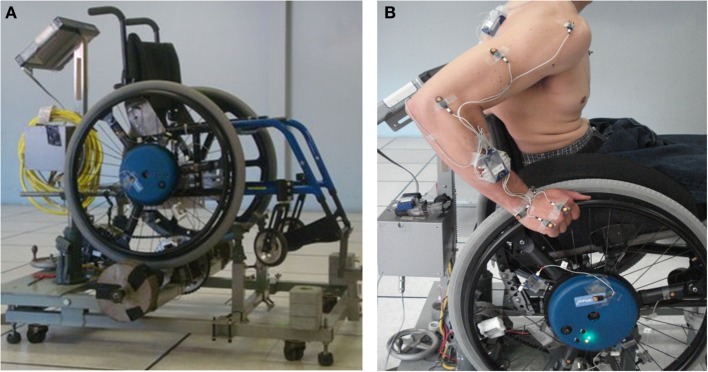
**Experimental setup: (A)** Manual wheelchair ergometer consisting of supporting frame, controlling computer and split rollers. **(B)** Subject on ergometer with markers affixed to the body and wheel.

Participants propelled their wheelchair on a stationary ergometer (Figure 1) during three conditions (e.g., Lighthall-Haubert et al., 2009): level propulsion at their self-selected speed (free), level propulsion at their fastest comfortable speed (fast), and propulsion on an 8% grade at their level self-selected speed (graded). Subjects acclimated to each condition until they felt comfortable (at least 30 s of propulsion) and a 10-s trial was recorded for each condition. Data were collected separately from both the dominant and non-dominant sides, with the side tested first randomly selected. Three-dimensional handrim kinetics were collected using an instrumented handrim (SmartWheel; Three Rivers Holdings, Mesa, AZ, USA). Trunk, ipsilateral upper extremity, and wheel kinematics were collected using a CODA motion analysis system (Charnwood Dynamics Ltd., Leicestershire, UK) with 15 active markers placed on landmarks on the body and the wheel (Figure 1).

### Data processing

Kinematic and kinetic data were processed in Visual3D (C-Motion, Inc., Germantown, MD, USA) using a low-pass, fourth-order, zero-lag Butterworth filter with cutoff frequencies of 8 and 10 Hz, respectively. A threshold of 1 Nm for the moment about the wheel axle was used to indicate the beginning and end of the push and recovery phases. Shoulder plane-of-elevation, shoulder elevation angle, shoulder internal-external rotation, elbow flexion-extension, and forearm pronation-supination angles were determined in accordance with International Society of Biomechanics recommendations (Wu et al., 2005). Range of motion values (ROMs) for these angles, peak and average tangential and resultant forces, fraction of effective force, cycle time, push percentage, and push angle were then calculated for each cycle and averaged across cycles for each subject during each condition (Table 1).

**Table 1:** **Definition of variables**.

Variable name	Abbreviation	Calculation
Range of motion	ROM	Maximum angle–minimum angle
Propulsion moment (about wheel axle)	*M_z_*	Direct Smart Wheel output
Anterior force	*F_x_*	Direct Smart Wheel output
Superior force	*F_y_*	Direct Smart Wheel output
Lateral force	*F_z_*	Direct Smart Wheel output
Handrim radius	*r*	Measurement
Tangential force	*F*_tan_	$\frac{M_{\text{z}}}{r}$
Resultant force	*F*_tot_	$\sqrt{F_{x}^{2}+F_{y}^{2}+F_{z}^{2}}$
Fraction of effective force	FEF	$\frac{F_{\tan}}{F_{\text{tot}}}$
Cycle time	CT	Based on *M_z_* thresholds
Push time	PT	Based on *M_z_* thresholds
Push percentage	PP	$\frac{\mathrm{PT}}{\mathrm{CT}}$
Push angle	θ	Angle between the positions of the hand at the start and end of the push phase (see Figure 2)
Number of loops	nloops	Based on the number of curve intersections
Signed area of the *i*th loop	*A_i_*	Surveyor’s formula (e.g., Braden, 1986)
Net radial thickness	NRT	$\frac{\sum_{i=1}^{\text{nloops}}A_{i}}{r\theta }$
Total radial thickness	TRT	$\frac{\sum_{i=1}^{\mathrm{nloops}}|A_{i}|}{r\theta }$

**Figure 2:**
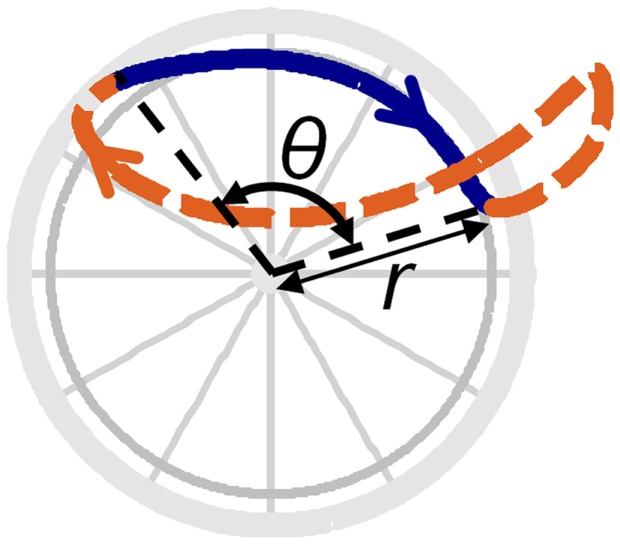
**Hand pattern variable definitions**. The solid line denotes the hand path during push phase, while the dashed line denotes the hand path during recovery phase. The handrim radius is denoted by the variable *r* and push angle is denoted by θ.

In addition, the third metacarpophalangeal joint center (MCP3) was located using a previously described method (Rao et al., 1996), and the MCP3 path was projected onto the plane of the handrim and averaged across cycles, resulting in a closed curve that details the full-cycle hand path or hand pattern (e.g., Figure 2; Boninger et al., 2002). Two objective, quantitative parameters were then calculated to characterize the hand pattern: net radial thickness (NRT) (a measurement of the displacement of the hand above the handrim) and total radial thickness (TRT) (a measurement of the distance between the hand and the handrim). For a detailed description of these parameters (NRT, TRT), see Slowik et al. (under review).

### Statistical analyses

To determine if there was asymmetry in any of the measured variables, statistical analyses were performed in SPSS (IBM Corp., Armonk, NY, USA) using two-factor (condition, side) repeated measures ANOVAs with a Huynh–Feldt correction in the case of non-sphericity. The condition factor consisted of three levels (free, fast, and graded) and the side factor consisted of two levels (dominant and non-dominant). If there was a significant interaction effect, pairwise comparisons were performed using paired *t*-tests with a Bonferroni adjustment for multiple comparisons. The unadjusted threshold for statistical significance for all analyses was set at α = 0.05. Condition main effects were not reported.

## Results

### Joint kinematics

Almost all significant side-to-side differences occurred in the kinematic variables (Table 2). Elevation plane ROM had a significant interaction effect, particularly due to a larger dominant side value in the graded condition (condition*side interaction effect, *p* = 0.006; graded, dominant to non-dominant pairwise comparison, *p* = 0.014). Elevation angle ROM was larger on the dominant side than the non-dominant side (side main effect, *p* = 0.015). Shoulder rotation ROM was larger on the dominant side, particularly due to a larger dominant side value in the graded condition (side main effect, *p* = 0.007; condition*side effect, *p* = 0.002; graded, dominant to non-dominant pairwise comparison, *p* < 0.001). Elbow flexion ROM was larger on the dominant side than the non-dominant side (side main effect, *p* = 0.044). Forearm pronation ROM had a significant interaction effect, particularly due to a larger dominant side value in the graded condition (condition*side effect, *p* < 0.001; graded, dominant to non-dominant pairwise comparison, *p* < 0.001).

**Table 2:** **Mean (SD) values for examined propulsion variables. D indicates a dominant side value and ND indicates a non-dominant side value**.

	Side	Free	Fast	Graded
**JOINT KINEMATICS**				
Elevation plane ROM (°) ∘■	D	72.6 (20.8)	81.3 (21.5)	85.7 (16.3)
	ND	72.4 (19.6)	81.6 (17.7)	81.8 (14.5)
Elevation angle ROM (°) ▴	D	22.8 (7.2)	22.7 (7.8)	19.8 (7.6)
	ND	21.7 (7.5)	21.7 (7.0)	18.8 (7.1)
Shoulder rotation ROM (°) ▴∘■	D	67.9 (22.5)	73.9 (21.5)	77.5 (17.3)
	ND	64.2 (23.1)	70.8 (21.1)	69.5 (19.0)
Elbow flexion ROM (°) ▴	D	45.7 (14.7)	52.7 (15.8)	60.3 (16.1)
	ND	44.2 (16.2)	51.1 (16.0)	57.7 (16.9)
Forearm pronation ROM (°) ∘■	D	28.8 (10.5)	32.0 (12.6)	36.9 (15.1)
	ND	28.9 (11.1)	31.4 (11.1)	32.4 (13.5)
**HANDRIM KINETICS**				
Average total force (N)	D	29.9 (7.7)	44.2 (13.1)	80.7 (18.1)
	ND	29.5 (7.8)	43.4 (12.6)	80.7 (19.7)
Average tangential force (N)	D	21.1 (5.3)	30.3 (7.8)	67.3 (13.9)
	ND	20.7 (5.3)	29.3 (7.3)	66.7 (14.7)
Peak total force (N)	D	45.2 (14.0)	77.7 (28.1)	127.1 (31.9)
	ND	44.6 (14.2)	74.8 (27.3)	127.1 (34.9)
Peak tangential force (N)	D	33.3 (10.5)	54.8 (16.4)	109.4 (26.2)
	ND	33.0 (10.5)	52.3 (15.5)	108.7 (26.6)
Fraction of effective force (%)	D	72.0 (11.4)	70.3 (10.7)	84.3 (9.5)
	ND	71.5 (11.0)	68.9 (9.8)	83.9 (9.7)
**SPATIOTEMPORAL VARIABLES**				
Cycle time (s)	D	1.15 (0.25)	0.78 (0.18)	0.79 (0.19)
	ND	1.12 (0.25)	0.77 (0.16)	0.78 (0.19)
Push percentage (% cycle)	D	36.0 (5.4)	32.0 (4.6)	55.6 (4.8)
	ND	35.5 (4.6)	31.9 (4.4)	55.2 (4.6)
Push angle (°) ∘■	D	74.9 (15.5)	79.8 (14.5)	85.4 (14.9)
	ND	73.4 (16.2)	80.2 (13.9)	84.0 (15.4)
NRT (m)	D	−0.016 (0.055)	0.013 (0.049)	0.011 (0.023)
	ND	−0.012 (0.053)	0.010 (0.047)	0.011 (0.021)
TRT (m)	D	0.051 (0.038)	0.051 (0.035)	0.021 (0.019)
	ND	0.048 (0.039)	0.050 (0.030)	0.021 (0.014)

▴ *denotes a significant side main effect*.

∘ denotes a significant condition*side interaction effect.

■ *denotes a significant dominant to non-dominant pairwise comparison in the graded condition*.

There were no other side main effects or interaction effects, and all differences were <5° except for shoulder rotation ROM during the graded condition (8°).

### Handrim kinetics

There were no significant side main effects or interaction effects in any of the kinetic variables.

### Spatiotemporal variables

Push angle had a significant interaction effect, particularly due to a larger dominant side value in the graded condition (condition*side effect, *p* = 0.025; graded, dominant to non-dominant pairwise comparison, *p* = 0.033). There were no other significant side main effects or interaction effects in the spatiotemporal variables.

## Discussion

The results suggest that low levels of asymmetry may exist in manual wheelchair propulsion, and that these levels may increase in the graded condition when the demand on the upper extremity is increased. However, we did not find any statistically significant side-to-side differences in any of the kinetic variables, and only one spatiotemporal variable (push angle) showed a significant side-to-side difference. We did find significant side-to-side differences in the joint ROMs, with dominant side values larger than those of the non-dominant side. However, the mean differences were small, with only one difference being larger than 5°. In addition, side-to-side differences were often smaller than differences between individuals or between conditions. Thus, while the comparisons showed statistical significance, the clinical significance of these differences is likely not high.

The magnitudes of the side-to-side differences were similar to those reported by others. An early study investigating racing propulsion (Goosey and Campbell, 1998) reported a non-significant mean difference of approximately 2° in the elbow flexion ROM in a sample of seven experienced wheelchair users. Others investigated standard handrim propulsion and reported non-significant mean differences of <1 N in peak and average handrim forces in 20 experienced wheelchair users (Koontz et al., 2001). Another group performed a series of three studies (Hurd et al., 2008a,b, 2009), examining side-to-side differences in kinetic and temporal variables for standard handrim propulsion on different terrains (12–14 experienced wheelchair users). All studies showed similar magnitudes of differences to the present study. Using similar statistical methods (i.e., repeated measures ANOVAs and/or paired *t*-tests), Hurd et al. (2008b, 2009) found only a single significant side-to-side difference (in average instantaneous power for propulsion on aggregate concrete). The third study (Hurd et al., 2008a) utilized a symmetry index and suggested that statistically significant levels of asymmetry were present for all investigated variables and terrains.

The lack of consensus regarding symmetry differences is likely due to a combination of different sample sizes and statistical methods. The present study may have been able to find statistical significance where others had not due to the large sample size (*n* = 80). In addition, while symmetry indices have been utilized in the study of gait (e.g., Sadeghi et al., 2000) and have potential in the analysis of manual wheelchair propulsion, the particular symmetry index used by Hurd et al. (2008a) disregarded the direction of asymmetries by taking the absolute value of observed differences, which resulted in only positive values. The difference between their symmetry index and the one that they attempted to replicate (Kaufman et al., 1996) may have led to an overestimation of the across-subjects mean levels of asymmetry. It is unlikely that dominant side data will be identical to the non-dominant side data for any single subject, and any small side-to-side differences that otherwise may have been neutralized across subjects (including those due to normal levels of experimental uncertainty and motion variability) were instead preserved by examining the absolute value.

The results of this study, in combination with previous results in the literature, suggest that the assumption of symmetry is reasonable when analyzing wheelchair propulsion in groups of subjects without secondary injury or pain in their upper extremities. However, our study only included data from subjects with paraplegia, so our conclusions may not be generalizable to other patient populations. A previous study found more asymmetry in propulsion biomechanics in individuals with multiple sclerosis than in individuals with spinal cord injury and able-bodied subjects (Fay et al., 2004), thus reinforcing the need to consider symmetry in the context of specific populations. In addition, it may not be appropriate to assume symmetry in the study of individual subjects as larger asymmetries may be present in individual subject data than in group-averaged data (e.g., Koontz et al., 2001; Schnorenberg et al., 2014), a finding that is also confirmed in our data. Individuals were found to have larger asymmetries than the group average. Studies should also be careful in assuming symmetry for propulsion during more strenuous conditions as we found the largest levels of asymmetry in the graded condition and a previous study concluded that asymmetry increased when propelling over outdoor terrain compared to laboratory terrain (Hurd et al., 2008a).

A potential limitation of this study is that only one instrumented wheel was used during data collection. As a result, dominant and non-dominant variables were recorded during separate trials. However, potential systematic differences between trials (e.g., fatigue effects) were minimized by randomly selecting the trial order. While any remaining systematic differences between trials could lead to overestimation of asymmetry, we still only found low levels. In addition, the alternative of using two instrumented wheels during a single trial is not without its own limitations. The side-to-side mean differences in kinetic variables that we observed were smaller than the documented accuracy and precision of instrumented wheels (e.g., Cooper et al., 1997; Wu et al., 1998; Guo et al., 2011). Even after calibration, there can be small differences between measurements from individual wheels, which are supported by a recent study that found differences between individual measurement wheels during a single trial were larger than single wheel differences between trials (Vegter et al., 2013b).

Another potential limitation is that subjects did not propel the wheelchair overground, but instead used a stationary ergometer set up to replicate overground propulsion. Although ergometers are unable to perfectly replicate overground propulsion, they do provide controlled conditions for data collection and have been shown to produce steady-state propulsion mechanics consistent with overground data (e.g., Koontz et al., 2001). However, propulsion on an ergometer is less constrained compared to overground propulsion. While the average power delivered to each handrim must be equivalent during straight-line overground propulsion, no such steering requirement exists for ergometer propulsion (e.g., de Groot et al., 2005). However, this limitation would likely lead to an overestimation of asymmetry, so the use of an ergometer likely did not alter the study conclusions.

In summary, our results support the assumption of symmetry in manual wheelchair propulsion for studies that analyze groups of subjects without significant upper extremity pain or impairment. Small asymmetries likely exist in propulsion variables, and these may increase when propelling under more strenuous conditions. Thus, the validity of the symmetry assumption should be carefully considered in light of the specific research aims and methods.

## Conflict of Interest Statement

The authors declare that the research was conducted in the absence of any commercial or financial relationships that could be construed as a potential conflict of interest.
